# An Unexpected Water Channel in the Light-Harvesting Complex of a Diatom: Implications for the Switch between Light Harvesting and Photoprotection

**DOI:** 10.1021/acsphyschemau.4c00069

**Published:** 2024-08-21

**Authors:** Vangelis Daskalakis, Sayan Maity, Ulrich Kleinekathöfer

**Affiliations:** † Department of Chemical Engineering, University of Patras, Caratheodory 1, University Campus, Patras, GR265 04, Greece; ‡ School of Science, Constructor University, Campus Ring 1, 28759 Bremen, Germany

**Keywords:** diatoms, molecular dynamics, water channel, protonation events, photosynthesis, photoprotection, carotenoids

## Abstract

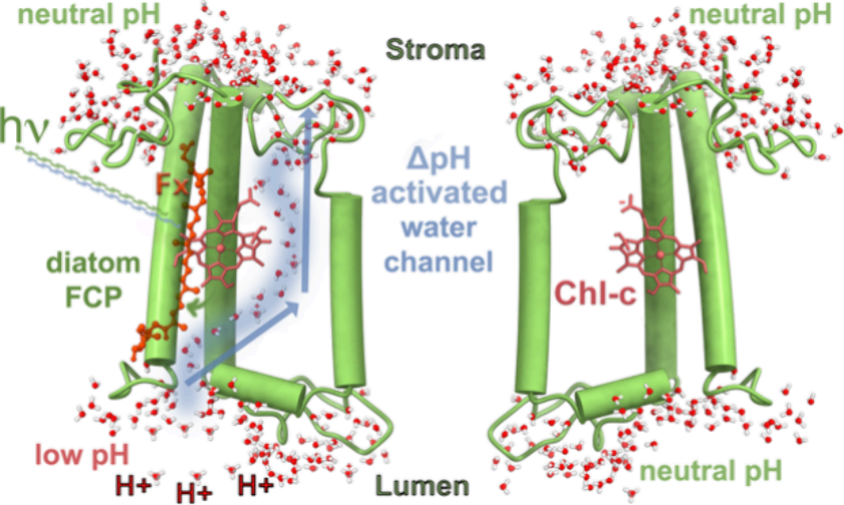

Many important processes in cells depend on the transfer of protons through water wires embedded in transmembrane proteins. Herein, we have performed more than 55 μs all-atom simulations of the light-harvesting complex of a diatom, i.e., the fucoxanthin and chlorophyll a/c binding protein (FCP) from the marine diatom *Phaeodactylum tricornutum*. Diatoms are unique models to study natural photosynthesis as they exert an efficient light-harvesting machinery with a robust pH-dependent photoprotective mechanism. The present study reports on the dynamics of an FCP monomer, a dimer, and a tetramer at varying pH values. Surprisingly, we have identified at low pH a water channel across FCP that selectively hydrates and protonates the acrylate of a Chl-c2 pigment located in the middle of the membrane. These results are further supported by QM/MM calculations and steered MD simulations on the proton dynamics. It is shown that proton hopping events between the lumenal and stromal sides of the membrane through the observed water channel are highly disfavored. This hindrance is due to the presence of residues Arg31 and Lys82 close to the acrylate, along with an hydronium desolvation penalty that shows close similarities to the water conductance in aquaporins. Furthermore, we provide strong evidence that this identified water channel is governing the transition between light-harvesting and photoprotective states of the major FCP complex in the diatom *P. tricornutum*.

## Introduction

Proton transport through proteins has been associated with the formation and exploitation of proton gradients in cells. Water molecules and polar side chains of residues can form “wires” within proteins, along which protons can be conducted in a controlled manner. This proton transfer becomes crucial for many bioenergetic functions that include the control of the transmembrane pH gradient as well as signaling pathways.^1^ Often, the transport of protons takes place along well-defined pathways that are enabled depending on the environment or the mechanism of action. Despite the Grotthuss mechanism of proton transfer in water, the formation of water wires in proteins does not automatically lead to a proton transfer through these water molecules and a high degree of proton selectivity can be maintained.^2^ Key examples include the cytochrome *c* oxidase of the cellular respiratory chain^3,4^ and aquaporins,^5−7^ both containing water channels but being able to maintain a transmembrane proton gradient (ΔpH). Apart from cellular respiration, for the proper function of reaction centers in photosynthetic systems, proton transfer therein is, of course, known to be important. Furthermore, a transmembrane ΔpH regulates the function of several light-harvesting complexes (LHCs) in photosynthesis especially in plants and algae and triggers the LHCs to switch between different states: a light-harvesting and a photoprotective one.^8,9^ Although photosynthesis is abundant and sustains most life and primary biomass production on earth,^10,11^ so far, no water wires or channels have been identified within LHCs.

Diatoms are ideal model organisms for the study of photosynthetic processes, and they are considered as primary producers of high importance in marine ecosystems like oceans, lakes, rivers, and streams.^12^ These unicellular, photosynthetic algae can rapidly adjust their light-harvesting properties and photochemistry within the thylakoid membranes of their chloroplast by modulating the transthylakoid membrane proton gradient.^12^ Both the diurnal cycle and the environmental conditions, e.g., turbulent ocean surfaces, induce fluctuations in the light intensity or spectral properties reaching the LHCs of diatoms.^13^ Thus, a robust mechanism that regulates transmembrane ΔpH and fast (de)protonation events of key residues within the photosynthetic systems is necessary to enable diatoms to adapt rather swiftly under fluctuating light conditions.^14−20^

**Figure 1:**
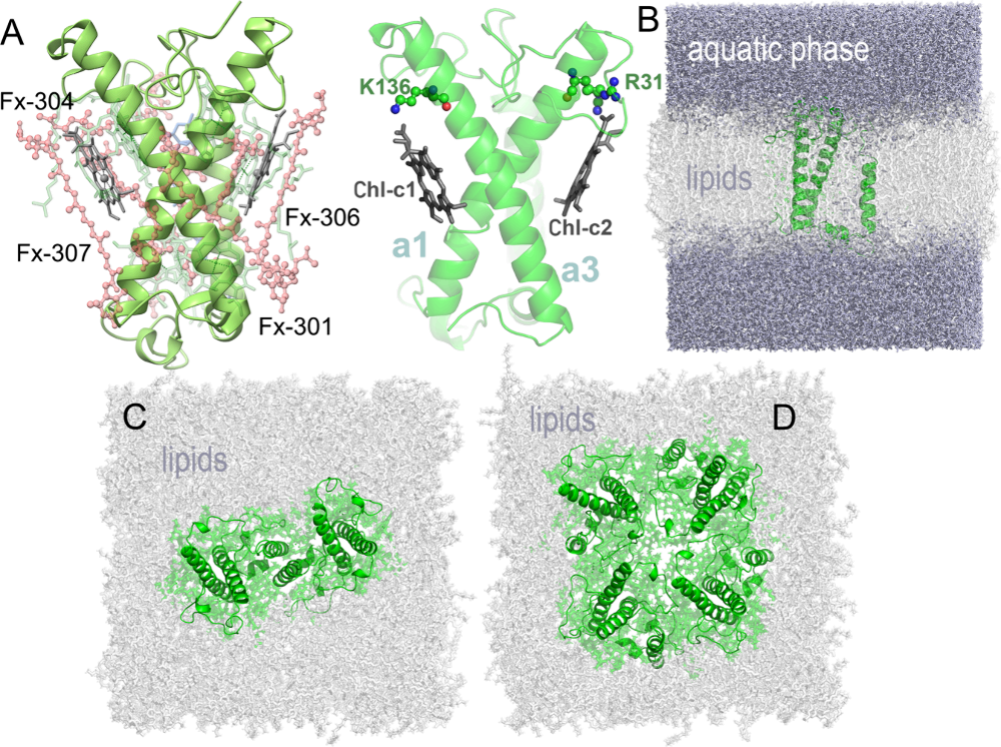
(A) Crystal structure of a monomer FCP from the diatom *Phaeodactylum tricornutum* (pdb: 6a2w). The polypeptide chain is depicted in green cartoon representations. Chlorophyll a (Chl-a) molecules are shown in faded green stick representations, selected fucoxanthins are labeled and shown in red ball and stick, and the two chlorophyll-c pigments (Chl-c1 and c2) are shown in gray. On the right, we show only the Chl-c1 and c2 chromophores of the whole pigment network for reference. Also shown are selected residues in a hydrogen bond distance to the acrylate of Chl-c1, i.e., Lys-136 (K136), as well as to the acrylate of Chl-c2, i.e., Arg31 (R31). Moreover, the helices a1 (residues 25–56) and a3 (residues 129–161) are marked for reference. (B) Side view of the FCP monomer embedded in a thylakoid membrane patch. Lipids are drawn as white licorice moieties and the water phases using dense blue-white sticks. (C, D) Top views of the FCP dimer and tetramer models embedded in thylakoid membrane patches. The lipids are depicted as white licorice moieties, while the water molecules have been omitted for clarity.

Diatoms express fucoxanthin (Fx) and chlorophyll a/c binding proteins (FCP) as their LHCs. The FCPs exert exceptional light-harvesting capabilities in the blue-green region of light, which is dominantly available under water (450–550 nm). Each FCP monomer from the diatom *Phaeodactylum tricornutum* (dimeric FCP) in the structure of Wang et al.^16^ binds seven Chls-a, two Chls-c, seven Fxs within its protein scaffold, and possibly one diadinoxanthin (Ddx) on the periphery. Thus, compared to the LHCs of higher plants, a considerably lower Chl-to-carotenoid ratio is found in FCPs.^16,21^ The availability of additional FCP structures, like the ones from the diatom *Chaetoceros gracilis* (tetrameric FCP-A, monomeric FCP-D, E, F), from *Thalassiosira pseudonana* (homodimeric and heterodimeric, monomeric)^22^ has revealed a remarkable variability in the structures of the FCP complexes with different numbers of pigments and varying multimerization states (monomers, dimers, oligomers, and flexible peripheral trimers).^16,21,23−26^ So far, only recently first studies on the exciton dynamics within FCP complexes were reported, which mainly focus on the FCP monomer without an extensive conformational sampling of multimeric states at the atomic scale.^18,20,27−29^ Furthermore, the protonation state of the acrylate of Chl-c pigments within FCP complexes^30−32^ (Figure 1A) has been proposed to be important in the transition between the light-harvesting (neutral lumenal pH) and the photoprotective (low lumenal pH) state.^20,33^ It seems, however, quite peculiar to consider (de)protonation of Chl-c acrylates, which are buried within the hydrophobic environment of the thylakoid membrane (Figure 1B). In the present study, we analyze the atomic-scale structural dynamics of monomeric, dimeric, and tetrameric FCPs reconstructed based on the crystal structure from the diatom *P. tricornutum*.^16^ The latter structure contains two Fxs Fx-301 and Fx-302, which are also present only in the dimeric^22^ and tetrameric FCPs^16,21,25^; thus, we have chosen not to probe trimeric units where these Fxs might be lost due to steric hindrance.^22^ Fx-301 has been associated with the transition between light-harvesting and photoprotective states in *P. tricornutum*.^18,34^ To this end, a putative water channel is found within the FCP protein scaffold that hydrates and potentially allows for (de)protonation of the Chl-c acrylates, an important insight that is unique for LHCs. Furthermore, this water channel is directly associated with an excitonic-based molecular switch that controls the transition between light-harvesting and photoprotective states of the FCP complex.

## Results and Discussion

### FCP Conformations

Monomer, dimer, and tetramer models of the FCP protein were built (Figure 1B–D) based on initial atomic coordinates from the FCP structure resolved from the diatom *P. tricornutum* (pdb: 6a2w).^16^ For more details, please refer to Experimental Methods section. Different protonation states for key residues were chosen to simulate varying pH states of the FCP complexes for this family of proteins.^15,19^ Two pH values were simulated for all FCP polypeptide chains: neutral pH 7.0 with all Asp and Glu residues treated as deprotonated and pH 5.5 with the lumen-exposed residues Glu-54, Glu-72, Glu-82, and Glu-158 as well as Asp-64 constantly protonated throughout the dynamics in line with proposals in the literature for the switch between the light-harvesting and photoprotective states.^16,19^ The later protonation states agree also with predictions of the PDB2PQR server, i.e., the PROPKA 3.0 method.^35^ For the acrylate groups of the Chl-c molecules, two protonation states were chosen: deprotonated at neutral pH and, according to recent studies,^20,33^ either protonated or deprotonated at pH 5.5 termed pH 5.5 and pH 5.5*, respectively. The protonation states have been considered according to *a priori* predicted p*K*_a_ values and do not refer to constant pH simulations. The residues His-39 and His-157 were protonated at the N_δ_ sites participating in hydrogen bonding interactions so that the crystal structure geometry is preserved around these residues. Nine FCP models were thus considered in total: monomers, dimers, and tetramers of FCP at neutral pH, at pH 5.5* and 5.5. The (*) in pH 5.5* marks that only lumen-exposed residues are kept protonated in the FCP polypeptides, whereas the acrylates of the Chl-c pigments remain deprotonated. Molecular dynamics (MD) trajectories were initiated based on these models. The models were simulated in numerous independent trajectories, i.e., replicas (*see* details in Experimental Methods section). The different pH values mimicked by the different protonation states simulate the light-harvesting (neutral pH) and the photoprotective conditions (lower pH values) for the FCP systems.^19^

**Figure 2:**
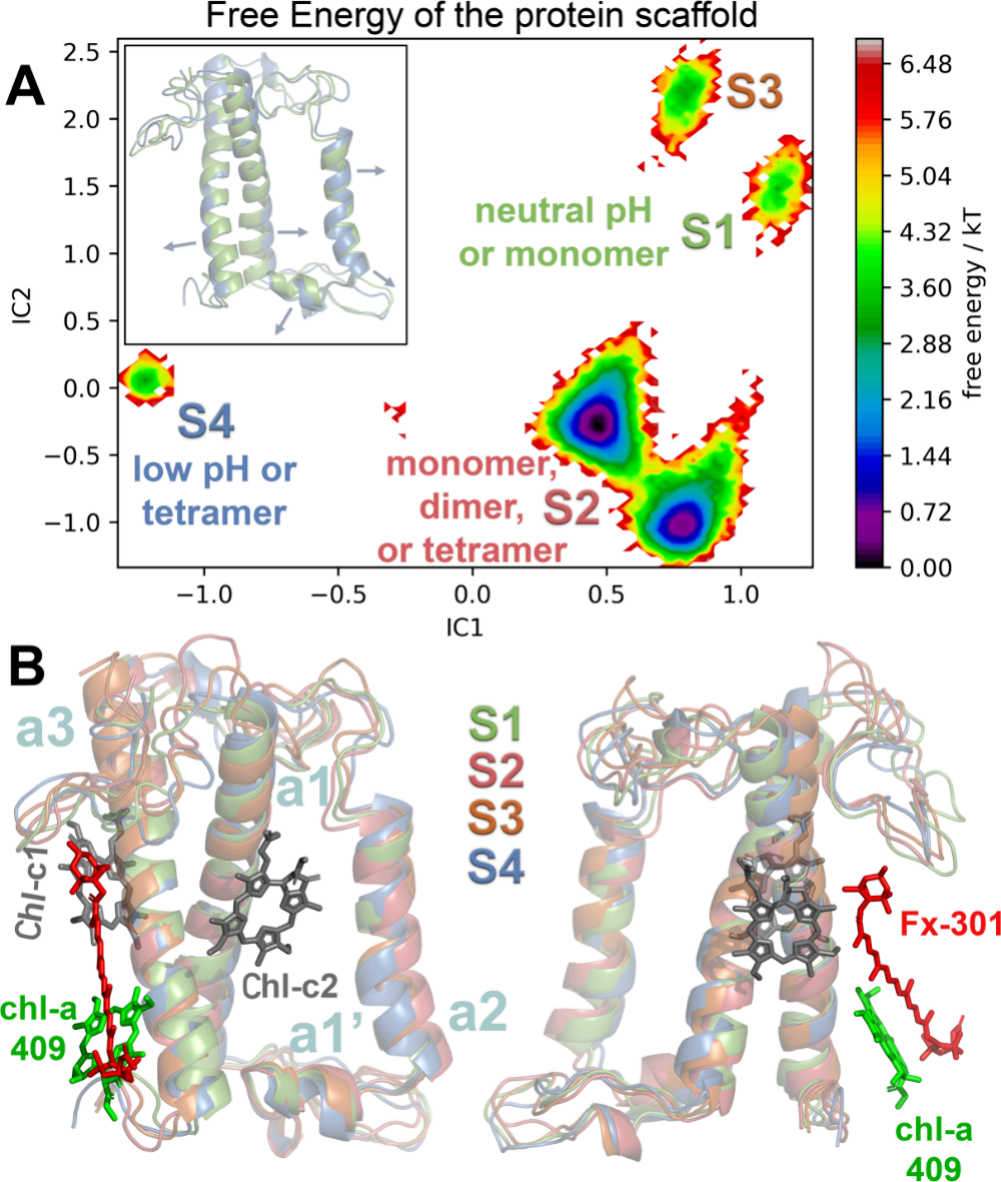
(A) Free energy of the protein scaffold spanned over the tICA components IC1 and IC2. The macrostates S1 to S4 are highlighted with labels denoting the qualitative contribution of each different state (monomer, dimer, tetramer, neutral-low pH) to the FES. The energy values are given in units of *k*_B_*T* with k_B_ being the Boltzmann constant and *T* the temperature. The configurational changes from S1 (green cartoons) to S4 (blue cartoons) are provided in the inset with arrows indicating the expansion of the protein scaffold. (B) FCP monomer in the different S1–S4 conformations color coded as in panel A. Selected pigments are also shown as superimposed from the crystal structure; Chls-c pigments are colored gray, Chl-a 409 green and the fucoxanthin carotenoid Fx-301 red. The FCP helixes are marked for reference (helix a1: residues 25–56, helix a1′: 75–83, helix a2:84–103, and helix a3:129–161).

The configurational space of FCP can be characterized by combining the all-atom MD simulations with Markov state modeling (MSM).^36−38^ To this end, the dynamics of individual monomers within the short MD trajectories of the monomer, dimer, or tetramer in the different states have been extracted (112 trajectories, 48 μs simulation time for individual monomers extracted from the monomer, dimer, and tetramer simulations). Herein, we first employ the time-structure-based independent component analysis (tICA) method to decrease the dimensionality of the configurational space explored over the MD trajectories and remove any redundant information. The reweighted free energy surface (FES) projected onto the configurational space of the FCP protein described by the two main tICA components IC1 and IC2 was determined based on the MSM analysis of all equilibrium trajectories, i.e., 48 μs of monomers. The result is shown in Figure 2A along with the position of the four identified macrostates S1 to S4. The four respective molecular FCP conformations of states S1 to S4 are depicted in Figure 2B. For reference, the qualitative contribution of each FCP state (pH, monomer, dimer, tetramer) to the FES representation in Figure 2A is indicated in Figure S1 in the Supporting Information (SI). The distinct conformations S1 to S4 are associated with different FCP states that represent macrostates along the slow dynamics of the FCP configurational space described by IC1 and IC2. It is remarkable that the FCP monomer dynamics correlates with those of the FCPs at neutral pH (state S1), whereas the tetramer dynamics aligns with those at low lumenal pH (S4), thus the quenched state, in correlation with a recent experimental study where the FCP-A tetramer is found severely quenched, via an ultrafast excitation energy transfer from Chl-a to Fx.^29^ States S1 and S4 are well separated in the chosen configurational space. State S2 is highly populated and shared between all pH and FCP multi- and monomeric configurations. The S2 state, thus, potentially is a transition state between the light-harvesting and the quenched state. Conformational differences between S1 and S4 are mainly observed for helices a1′ and a2, as shown in Figure 2B. We note that helices a1′ and a2 bind the Ddx carotenoid and reside at the interface of the two monomers in the FCP dimer resolved in the structure of Wang et al.^16^ Furthermore, the FCP helices a1′ and a2 are predicted to be also at the interacting interface (cross section) between FCP and the photoprotective LHCX1 family of proteins.^18^

### The Environment of the Chl-c Acrylate

**Figure 3:**
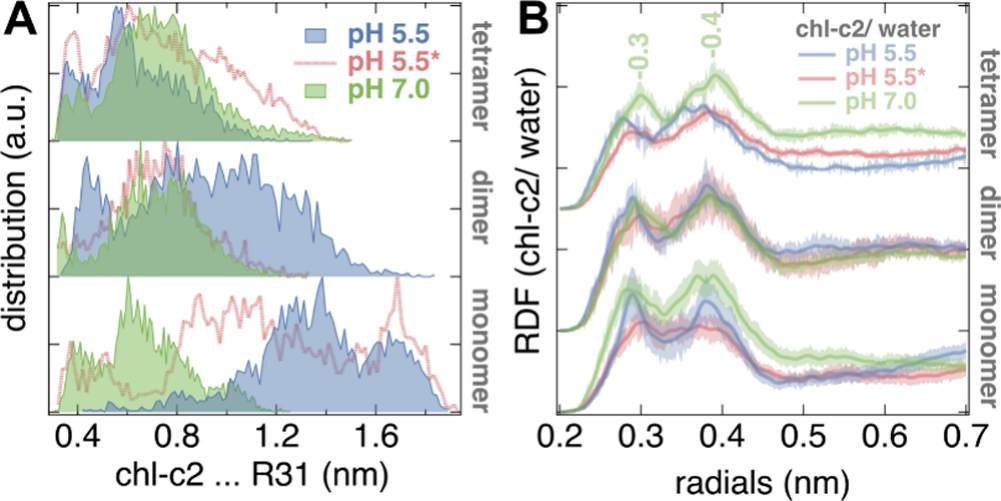
(A) Distributions of the distances between the acrylate of Chl-c2 and residue Arg31 (R31) for the different FCP states. (B) Radial distribution functions (RDFs) of the water molecules around the Chl-c2 acrylate averaged over 500 ns windows and over the monomers in each dimer or tetramer trajectory. The colored shaded areas represent the standard deviations of the averaging.

The distribution of distances between the Chl-c2 acrylate (carboxyl oxygen) and Arg31 (sp3 carbon in the side chain) is shown in Figure 3A for the different FCP states. The respective distance distributions between the Chl-c1 acrylate (carboxyl oxygen) and Lys-136 (side chain nitrogen) is shown in Figure S2 in the SI. We observe a pH-, i.e., a protonation-dependent distribution of these distances in FCP. These latter distributions correlate with a recent computational study on the protonation state of the Chl-c acrylate.^20^ As a general trend and compared to the deprotonated case at pH 7.0, when the Chl-c acrylates are treated as protonated (pH 5.5), larger distances from Chl-c to Arg31 or to Lys-136 are found along the MD trajectories. Remarkably, large distances between the Chl-c acrylate and Arg31 or Lys-136 are also detected when only the lumen-exposed FCP residues are treated as protonated, even when the Chl-c acrylates are treated as deprotonated in the case termed “pH 5.5*” above. Thus, there must be a synergetic effect between the protonation states of the lumen-exposed residues in FCP and the acrylate protonation states. The effect of the acrylate protonation is more pronounced in the distributions of the Chl-c2–Arg31 distance and less pronounced for the Chl-c1–Lys-136 distances with the latter mainly depending on the protonation state of lumen-exposed FCP residues. As the pH is lowered in the lumenal space, the FCP complex expands (blue-white arrows in the inset of Figure 2A) compared to the neutral pH state (S1—green). This expansion is likely due to the protonation of the Chl-c acrylates, which is associated with weaker interactions and larger distances to Arg31 and Lys137 (Figures 1A–3A and Figure S2 in the SI).

### Water Channel in FCP and Structural Features

How can the acrylates of the Chl-c pigments switch between protonated and deprotonated states while being buried within the membrane? What is the connection of the protonation events to the different FCP states identified by the MSM scheme? By visual inspection of the numerous classical MD trajectories, we have identified an accumulation of water molecules mainly around the Chl-c2 acrylate in the different probed models.

**Figure 4:**
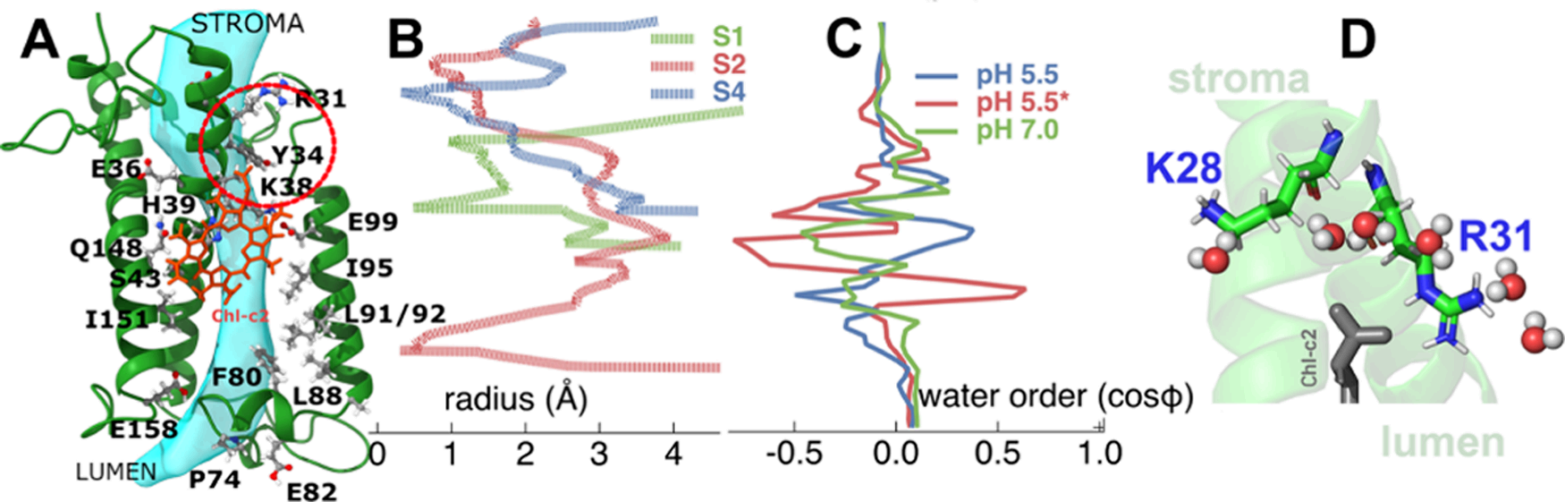
(A) The FCP monomer is shown in cartoon representations along with the Chl-c2 pigment. The path of the identified water channel is indicated by a large blue/mesh surface in a qualitative manner. Selected residues are shown as sticks along the channel. The “STROMA” and “LUMEN” labels indicate the positions of the respective aquatic phases. (B) Radius of the water channels along the *z*-coordinate across the thylakoid membrane (membrane normal) for the MSM-predicted FCP states. The *z*-axis has been replaced by the FCP protein for reference. (C) Order parameter of the water molecules along the membrane normal for the S1, S2, and S4 states. As in (B), the protein structure in panel A is provided as reference for the position along the membrane normal. (D) The residue Arg31 (R31) is shown to be in close contact with the acrylate of Chl-c2 along with an adjacent Lys28 (K28) on helix a2 of FCP. A wire of water molecules that change their orientations is also depicted out of the MD simulation. The associated region in the (A) FCP crystal structure is highlighted by a red dotted circle.

To further quantify our observations, we have calculated the distribution of water molecules around the carboxyl carbon of the Chl-c acrylates in terms of the radial distribution functions (RDFs) over the trajectories and the results are shown in Figure 3B for the Chl-c2 acrylate and Figure S3 in the SI for the Chl-c1 acrylate. Peaks at short radial distances of 0.3 nm are only visible for Chl-c2 for all pH and multimerization states that indicate a strong presence of water molecules only around the Chl-c2 acrylate. There is no such accumulation of water molecules around the Chl-c1 acrylate for the monomer and dimer simulations. A somewhat different picture emerges for the tetramer, where water molecules are also found close to the Chl-c1 acrylate at all protonation states (Figure S3 in the SI). Therefore, we cannot exclude a synergy that might emerge by the multimerization of FCP to enable hydration of the acrylates of both Chl-c1 and Chl-c2. Moreover, we quantified the water channel passing Chl-c2 by the program Hole 2.^39,40^ It is shown as a large blue mesh/surface in Figure 4A with an entrance at Glu-158/Phe-77 and formed roughly by residues Phe-80, Glu-82, Ala-85, Gly-86, and Leu-88 (lumen); Leu-91, Leu-92, Ile-95, and Glu-99 (core); and Tyr-34, Val-35, and Lys-38 (stroma). It is interesting to note that Leu, Ile, Phe, and Gly residues are commonly found lining up in the main pores of aquaporins as well.^5−7^

The radius of the water channel along the thylakoid membrane normal has been calculated^39,40^ and is presented in Figure 4B for the different MSM-predicted macrostates (state S3 has no identifiable channel). Only state S2 has a well-defined water channel that connects the lumen to the stromal aquatic phases, across the thylakoid membrane that passes by the Chl-c2 acrylate. States S1 and S4 exert rather shorter water paths from the stromal area to the Chl-c2 acrylate. It seems indeed possible that even if the Chl-c2 pigment is buried within the hydrophobic environment of the thylakoid membrane, the FCP protein accommodates a water channel along which a proton could migrate from the lumenal space toward the acrylate of Chl-c2, when the pH drops in the lumen space, i.e., under photoprotective conditions. The present data correlates with experimental evidence that a 3:1 ratio of protonated to deprotonated Chl-c pigments is present within FCP proteins,^33^ which might indicate different water channel openings depending on the observed states or that Chl-c1 mainly remains in the deprotonated state.

Equilibrium trajectories show a diffusive character of the water within the identified channel, described in Figure 4A–D. To better understand the flow of water, pressure was applied to the water molecules based on a method proposed in the literature.^41^ We thus have run short nonequilibrium 10 ns trajectories for the dimer in the neutral and low pH states with external forces applied to all water molecules with a force constant of 5000 kJ/mol·nm. At the same time, the membrane was restrained at its position by applying constant forces in the opposite direction on all heavy atoms of the thylakoid lipids and on the Cα atoms of the FCP. The water current, i.e., a directed and not diffusive motion, was determined by a linear fit to the water movement in the 10 ns-long simulations while the first 2 ns of the trajectory was disregarded to avoid transient effects before reaching a steady-state flux. Absolute values of the water currents are heavily dependent on the applied forces and thus are not listed. However, nonzero currents have been found for all different FCP protonation states (pH 7.0, 5.5*, 5.5). The largest current has been obtained for the pH 5.5* protonation state, about 1.5 times larger than that calculated for the pH 7.0 or 5.5 cases.

**Figure 5:**
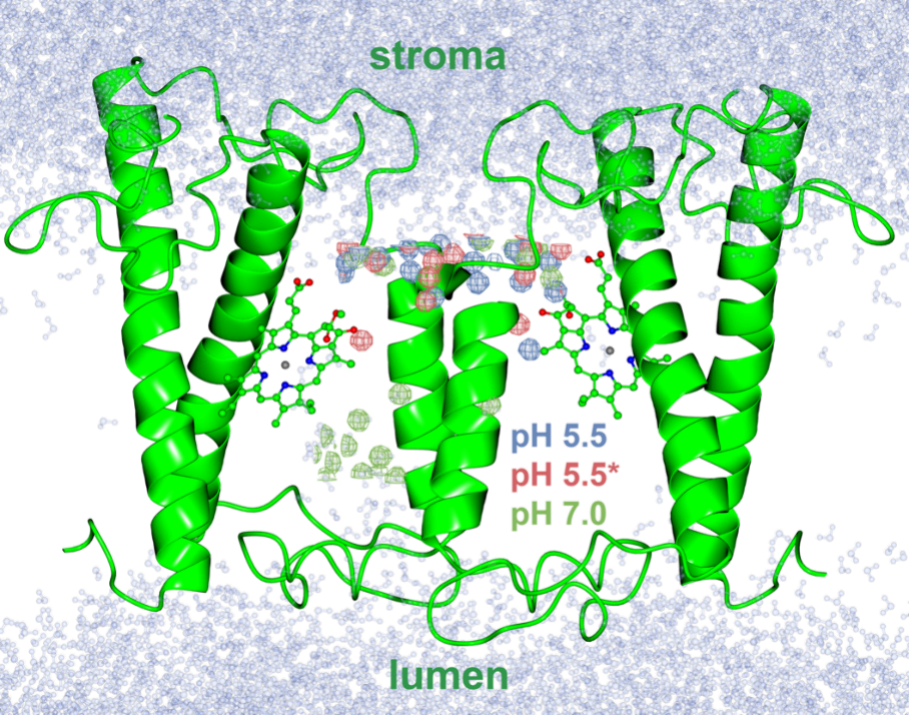
The FCP dimer from the X-ray crystal structure is shown in green cartoon along with the Chl-c2 pigments in solid ball and stick representations. The aquatic phases are shown in pale blue and transparent ball-and-stick representation from a snapshot of the pH 7.0 runs. The colored wired spheres represent waters with large scattering densities that indicate immobile waters at pH 5.5 (blue), pH 5.5* (red), and pH 7.0 (green).

An additional analysis has been performed employing an adapted methodology that can reproduce average electron density maps from crystalline MD (cMD) trajectories in an NVT ensemble.^42^ Here, we have not run any additional cMD trajectories but instead we based our analysis on the short nonequilibrium 10 ns NVT trajectories of the FCP dimer, as described previously. To this end, we have chosen the dimer model that matches the symmetry of the crystal structure from Wang et al.^16^ Although we cannot reproduce the actual X-ray diffraction data, as this would require a cMD setup, the results from this kind of analysis can add to the qualitative insight into the mobility of the water molecules within the FCP scaffold. Therefore, we employed the same analysis protocol as described in ref (42) by first computing mean structure factors at a resolution of 1.6 Å employing the xtraj.py in the Lunus software suite (http://github.com/lanl/lunus) and cctbx methods within the CCP4 suite of programs.^42−45^ An MD-derived water-only density (colored wired spheres in Figure 5) was determined or alternatively electron density maps by averaging structure factors and fast Fourier transform (FFT). Strong density peaks of the MD water molecules (at the wired spheres) show strongly interacting and immobile waters with high occupancy in the given positions. A high electron density, which indicates that the water structure is ordered, is found toward both the lumenal and stromal regions at pH 7.0 (green wired spheres), whereas a high electron density is observed for water molecules only toward the stromal side for the lower pH values (blue and red wired spheres). This finding indicates a pH-dependent distribution of (im)mobile waters. If we consider a water flow across the FCP scaffold, this should be inhibited at neutral pH in accordance also with Figure 4B where only a short partial channel is calculated at pH 7.0. The presence of immobile waters at the lumenal side for pH 7.0 indicates that strong protein–water interactions at that side (i.e., deprotonated Glu/Asp–water interactions) could prevent the flow of waters toward the stromal side. However, at lower pH values, water molecules at the lumenal side can move across the protein scaffold, indicated by the absence of immobile waters therein at pH 5.5(*). This water channel allows for a proton transfer from the acidified lumenal side toward the Chl-c2 acrylate, at the same time inhibiting the transport of that proton to the stromal side (*see also further analysis of the water orientations along the channel below*).

### Proton Conductance along the Water Channel in FCP

**Figure 6:**
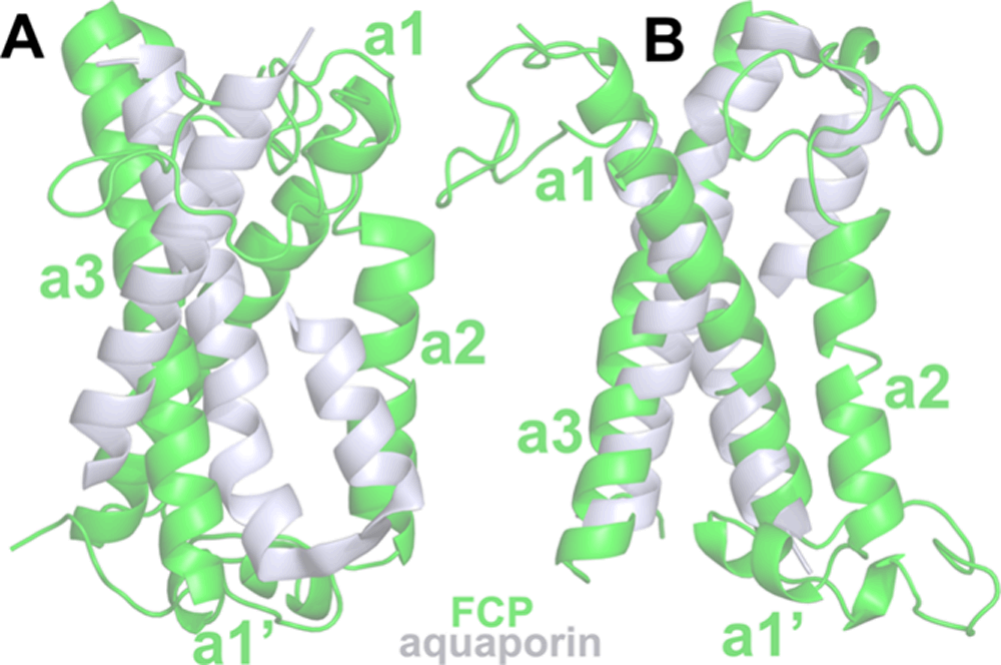
(A) FCP monomer (pdb: 6a2w, green) and selected helices of spinach aquaporin (pdb: 4ia4, grayish) superimposed in different orientations (A, B) to each other.

What could be the implications of such a water channel across the FCP light-harvesting complex? First, a water and with this a possible hydronium ion permeation across the thylakoid membrane would be detrimental to the buildup of a proton gradient used for ATP synthesis or for an enhanced ΔpH as a trigger for photoprotection.^46,47^ Water channels, similar to the one detected here, have been reported for aquaporins, where despite the presence of a water wire-like channel, protons cannot cross between the aquatic phases due to an exclusion mechanism, i.e., an orientational tuning.^48,49^ In fact, a superposition of the FCP helixes (pdb: 6a2w, residues 25–56,85–102,130–160)^16^ with those from an aquaporin in spinach (pdb: 4ia4, residues 160–184 and 222–263)^50^ employing the *super* module in PyMOL 2.5 (https://pymol.org/2/) shows a clear structural similarity (Figure 6). A common shorter transmembrane helix is present along with longer helices in both the FCP and aquaporin structures. For light-harvesting systems, this shorter transmembrane helix (a2 in FCP) is a unique feature of FCP and unlike what is found in higher plants.^51^

To probe the exclusion mechanism, i.e., the orientational tuning, in FCP, we have calculated the order of the water molecules across the identified water channel in FCP. The corresponding order parameter is given by the cosine of the angle φ formed between the dipole moment vector of the water molecules and the membrane normal.^52^ The values of cos(φ) have been averaged over all water molecules in bins along the *z*-axis, i.e., along the membrane normal, as shown in Figure 4C. A value of zero for this order parameter usually indicates that the water molecules are randomly orientated in the channel. Thus, this order parameter across the thylakoid membrane can be used to determine the influence of the protein pigments on the water molecules. Figure 4C depicts the order parameter for the different FCP states and the FCP monomer as reference, calculated from the short nonequilibrium trajectories (10 ns) mimicking a water pressure. At neutral pH (7.0), the waters within the channel become disordered, i.e., cos(φ) fluctuates around zero, whereas more order is induced for the low-pH states. The direction of the water molecules is practically reversed roughly in the middle of the channel for the pH 5.5* state, which is also associated with an open water channel across the FCP protein (Figure 4B). The presence of Arg31 close to the Chl-c2 acrylate, as well as Lys28,^16^ might be important (Figure 4D), as interruptions in the hydrogen-bonded water network have previously been observed in the presence of positively charged residues disfavoring proton hopping events between water molecules.^5,48^

Electrostatic interactions are thought to be the important elements that prevent proton permeation in aquaporins.^48,53−55^ The aquaporin homotetramer has been shown to act as a two-stage filter.^48^ The first stage of the filter is located at the center of the water channel and is the NPA (Asn-Pro-Ala) region.^48,54−56^ A strong barrier is observed at this site, which prevents the permeation of protons past NPA. This occurs due to the encounter of the positively charged residues, which have the ability to isolate a water molecule by forming a hydrogen bond with its oxygen.^48,54−58^ The residues of the NPA motif that extend into the constriction region form hydrogen bonds with water oxygen atoms and force its dipole moment to orient perpendicular to the channel axis, thus creating oriented water molecules in the pore.^48−58^ This orientation is further stabilized by the presence of hydrophobic residues along the pore (below also, see a comparison with FCP).^48−62^ Thus, orientational tuning is one factor that contributes to the inhibition of the proton translocation in aquaporins.^2^ However, complementary factors are also in place. An energy barrier has been associated with the loss of the H_3_O^+^ solvation energy upon its transfer from the bulk to the narrow region at the entrance of the channel and which increases toward the center of the pore.^63^ This increase is associated with the second stage, which is active at the extracellular surface of the channel in the aromatic/arginine (ar/R) constriction region.^60,62,53,48^ Upon the entrance to the channel, water–water hydrogen bonds need to be broken to allow a water molecule to pass through the narrow NPA region; however, the protein provides counter interactions, which largely compensate for the cost of breaking the water–water bonds.^48,60,66^ Protons located near the NPA region quickly leave the pore in either the extracellular or intracellular direction.^53^ From the intracellular side, protons reach the channel efficiently, while extracellular protons stick to the ar/R region for some time before leaving the channel.^53,57^ Disruption of the water wire interacting via hydrogen bonds either at the first or second stage is the main obstacle for protons to cross the channel.^58,53,55^ The aquaporin channels thus achieve their high-water permeability through a sophisticated tuning of hydrogen bonding networks.^48,60^ Therefore, the reasons for preventing protons from crossing the channel in aquaporins are mainly electrostatic in nature: (a) one is bipolar orientation of water; the central water molecule is prevented from forming hydrogen bonds with neighboring water molecules, thus disrupting the proton flow. A specific hydrogen bonding pattern is formed in the channel, and the orientation of the hydrogen bonds is disrupted at the center of the pore so that the protons of the water molecules are oriented outward. (b) Another is nonspecific solvation effects.^48−58,57−54^

**Figure 7:**
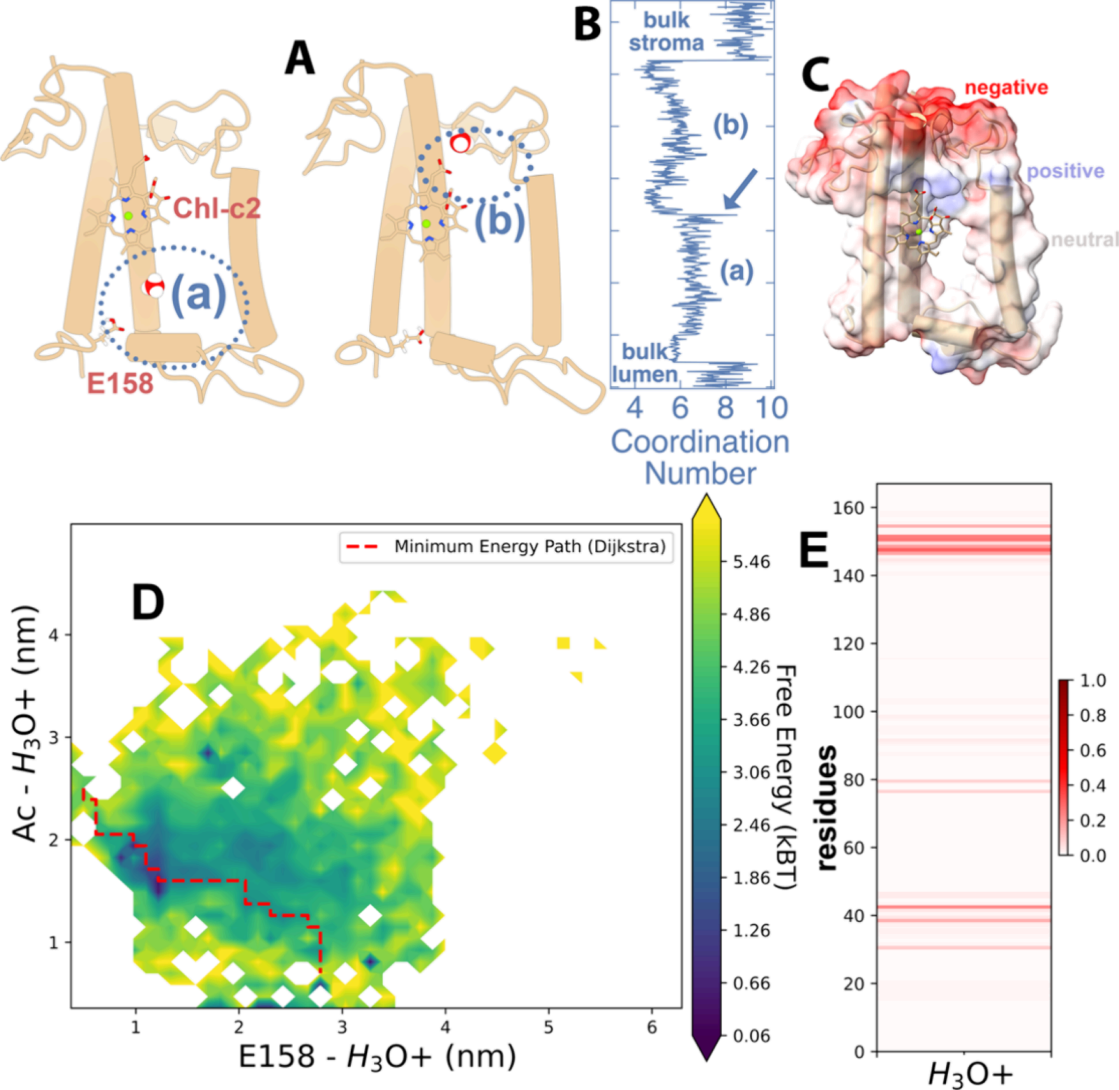
Steered MD simulation for an H_3_O^+^ along the identified water channel. (A) Two snapshots of the hydronium trajectory through FCP highlighting (a) a region before and (b) a region after the binding of H_3_O^+^ to the acrylate of the Chl-c2 molecule. The hydronium ion is shown with red oxygen and white hydrogen spheres. Glu-158 (E158) and Chl-c2 are also shown for reference. (B) Coordination number (solvation) for H_3_O^+^ along the trajectory including bulk and channel with regions (a) and (b) highlighted. A blue arrow indicates the binding of H_3_O^+^ to the Chl-c2 acrylate. The *y*-axis roughly indicates the position along the membrane normal in the scale of the structures in (A). (C) The electrostatic potential is superimposed on the FCP structure, indicating the negative (red), neutral (white), and blue (positive) regions. (D) Free energy of the H_3_O^+^ on two coordinates; the H_3_O^+^ distance to (i) the Chl-c2 acrylate (Ac, carboxyl group) and (ii) to the Glu-158 (E158) side chain carboxyl group. The energy values are given in units of *k*_B_*T* with *k*_B_ being the Boltzmann constant and *T* the temperature. A minimum energy pathway is shown as calculated by the Dijkstra algorithm. (E) Protein residues (horizontal axis) that interact with H_3_O^+^ associated with the energy surface of D. The color legend spans from increased strength and large residence times (dark red) to no interaction (white).

How do the abovementioned reasons apply to the FCP channel identified herein? For the FCP monomer model with deprotonated Chl-c2 acrylate, an additional MD simulation was run. Assuming an acidified lumen (protonated lumen-exposed Asp-Glu residues), a H_3_O^+^ molecule was put into the aquatic phase of the model and described by parameters given in the literature.^70^ After equilibration for 100 ns, it was steered^71^ through the channel past the Glu-158 (entrance of the channel) and toward the deprotonated Chl-c2 acrylate and the stromal space with a force constant of 1000 kJ/mol nm within about 37 ns (Figure 7). During the steering, the H_3_O^+^ coordination number was calculated with a 0.35 nm cutoff distance at the first minimum of the associated radial distribution function (please refer to the SI for the RDF in Figure S4). The coordination number was calculated, as described in the Plumed literature.^72^ We see a change in the coordination number as H_3_O^+^ enters the channel from the lumenal bulk (Figure 7B, blue line), which reflects a desolvation penalty (loss of solvation energy) for H_3_O^+^ to enter the narrow pore of the channel from the bulk. The coordination numbers slightly increase along the channel area (a) until the binding to the Chl-c2 acrylate (indicated by the blue arrow in Figure 7B). After H_3_O^+^ is dissociated from Chl-c2, we observe a further drop in the H_3_O^+^ coordination number along the channel area (b). Although the overall solvation of H_3_O^+^ is lower within the whole channel than in the bulk, the energy penalty becomes more significant beyond the Chl-c2 acrylate in region (b). The positive and neutral residues next to the Chl-c2 acrylate (electrostatic potential in Figure 7C) that are responsible for the orientational tuning can possibly also be responsible for the desolvation of H_3_O^+^ in that region; H_3_O+ would not coordinate the positively charged side chains in the area. This comes at odds with the aquaporin case, where positively charged residues can isolate a water molecule by forming a hydrogen bond with its oxygen. To further explore the configuration space of the H_3_O^+^ within the FCP protein scaffold, we performed random acceleration MD (RAMD)^73^ on 10 different structures extracted from the previous steered MD runs. The initial H_3_O^+^ positions were chosen around either E158, or the Chl-c2 acrylate (<0.25 nm) as the two binding sites, with 100 trajectories per case, resulting in 2000 trajectories (7.4 ns cumulative time). Based on the RAMD method, for each trajectory, a force (***F***, 585 kJ/mol) with random orientation is applied to H_3_O^+^; if H_3_O^+^ has moved beyond a set threshold (0.4 nm), the simulation is terminated, otherwise a new random direction of the force is generated. In this way, we mapped the configuration space of H_3_O^+^ within the FCP scaffold and calculated a FES based on the probability density distribution (−ln *P*(x)), as shown in Figure 7D. The minimum energy path of H_3_O^+^ from E158 to binding to the Chl-c2 acrylate is calculated using the Dijkstrat algorithm and is also indicated in Figure 7D by a dashed red line. It is interesting to note that at the end of the pathway (around Chl-c2 acrylate), there are high energy regions, or areas unexplored by the RAMD, indicating unfavorable regions for H_3_O^+^, in line with the desolvation barrier discussed above. We have also employed the Contact Map Explorer (https://github.com/dwhswenson/contact_map), which builds on the MDTraj tools (https://www.mdtraj.org/1.9.8.dev0/index.html) cumulatively for the 2000 RMAD generated trajectories to produce a protein–H_3_O^+^ contact map (Figure 7E). H_3_O^+^ is sampled either within the identified channel that matches the prediction by Hole 2.0 (Figure 4A and Figure S5A) where it is adequately hydrated, or in hydrogen bonding interaction with FCP residues as shown in Figure S5B. The positions of these key residues are shown in Figure 4A. Based on this hydrogen bonding network (Figure S5A,B), the water channel can indeed support proton transfer, as hydronium can be adequately coordinated by oxygen atoms of several residues along the protein scaffold. We note that the access of H_3_O^+^ to the stromal side is inhibited by unfavorable interactions.

Together with the orientational tuning described before, where a change in the direction of the water molecules would prohibit the leakage of protons (or hydronium ions) across the thylakoid membrane,^48,49^ the additional reduction in the solvation energy roughly at the same point as the orientational tuning inhibits the hydronium (or proton) translocation to the stromal area but can ensure the protonation of Chl-c2. Note that Glu-158 at the entrance of the channel can exchange a proton with nearby waters aiding in the structural diffusion and charge delocalization within Eigen H_9_O_4_^+^ or Zundel H_5_O_2_^+^ cations, altering the desolvation penalty for H_3_O+ for entering the channel.^70^ By acidification of the lumenal space, Arg31 moves away from the Chl-c2 acrylate and the acrylate is left deprotonated in the vicinity of waters (or H_3_O^+^) from where it grabs a proton. When lumen-exposed residues get protonated, Arg31 indeed moves away (even when the acrylate is still deprotonated, due to conformational changes, Figure 3A), a condition in which the Chl-c2 acrylate accepts a proton whenever and from wherever it can get one.

Water/proton channels are not that uncommon in photosynthesis. PSII catalyzes the water oxidation reaction in the oxygen-evolving complex (OEC), a heteronuclear Mn_4_CaO_5_ cluster. This requires the transport of lumenal water to the OEC and the simultaneous release of protons into the lumenal bulk water. In PSII, it is possible for protons to be transferred from the interior to the surface of the protein.^62,74−76^ Water molecules are transported through three main water channels (large, wide, and narrow around the OEC) to the OEC and are surrounded by hydrogen bond acceptors, whereas the formed water networks can transport protons, with the water molecules being firmly fixed at their positions.^62,74,75,77,78^ Water is oxidized at the inorganic OEC core, removing the produced protons into the lumen through the long water channel to the protein surface.^62,74,75^ Through five increasingly oxidized states, an oxygen–oxygen bond is formed and four protons are released.^62,74,75,77^ In detail, water channels and proton exit pathways have been proposed in the literature, namely, O1, O4, and Cl1, to ensure substrate (water) supply and release of products (O_2_ and H^+^).

There is a diversity between the different channels: O1 is wider with water molecules of higher mobility compared to the much narrower O4 and Cl1 channels where water is less mobile. It has been suggested that the supply of water and the release of protons through O1, O4, and Cl1 could be selective between different intermediates of Kok’s S_0_–S_4_ cycle and that the O4–Cl1 channels with less mobile water could be more suitable for proton transfer.^79^ Proton transfer occurs via the Grotthus mechanism.^62,75^ Water molecules and side chains of amino acids serve as hydrogen bond donors or acceptors so that the proton is displaced through hydrogen bonding networks.^62,75^ Thus, in these cases, protons are transported without hindrance.^62,75^ The Glu-65 (D1) residue in the Cl1 channel has been proposed as a gate and bottleneck for water and proton transfer in PSII. Conformational changes near the Glu-65 residue and disruption of an H-bond interaction with Gln-335 (D1) appear to be a crucial step in controlling this bottleneck around OEC in PSII.^79^ In this study, we have found, at the entrance of the FCP water channel, Glu-158 in an H-bond interaction with Gln-159 at about 0.27 nm at neutral pH and the crystal structure.^16^ However, upon acidification of the lumen and subsequent protonation of Glu-158, the latter distance becomes 0.78 nm in our MD equilibrium trajectories. The significant rotation of the Glu-158 side chain away from Gln-159 opens the water (proton) gate therein. Evolutionarily, this appears to be a well-established strategy for gating water molecules and protons through channels in PSII.

### Protonation Dynamics at the Chl-c2 Acrylate Site

**Figure 8:**
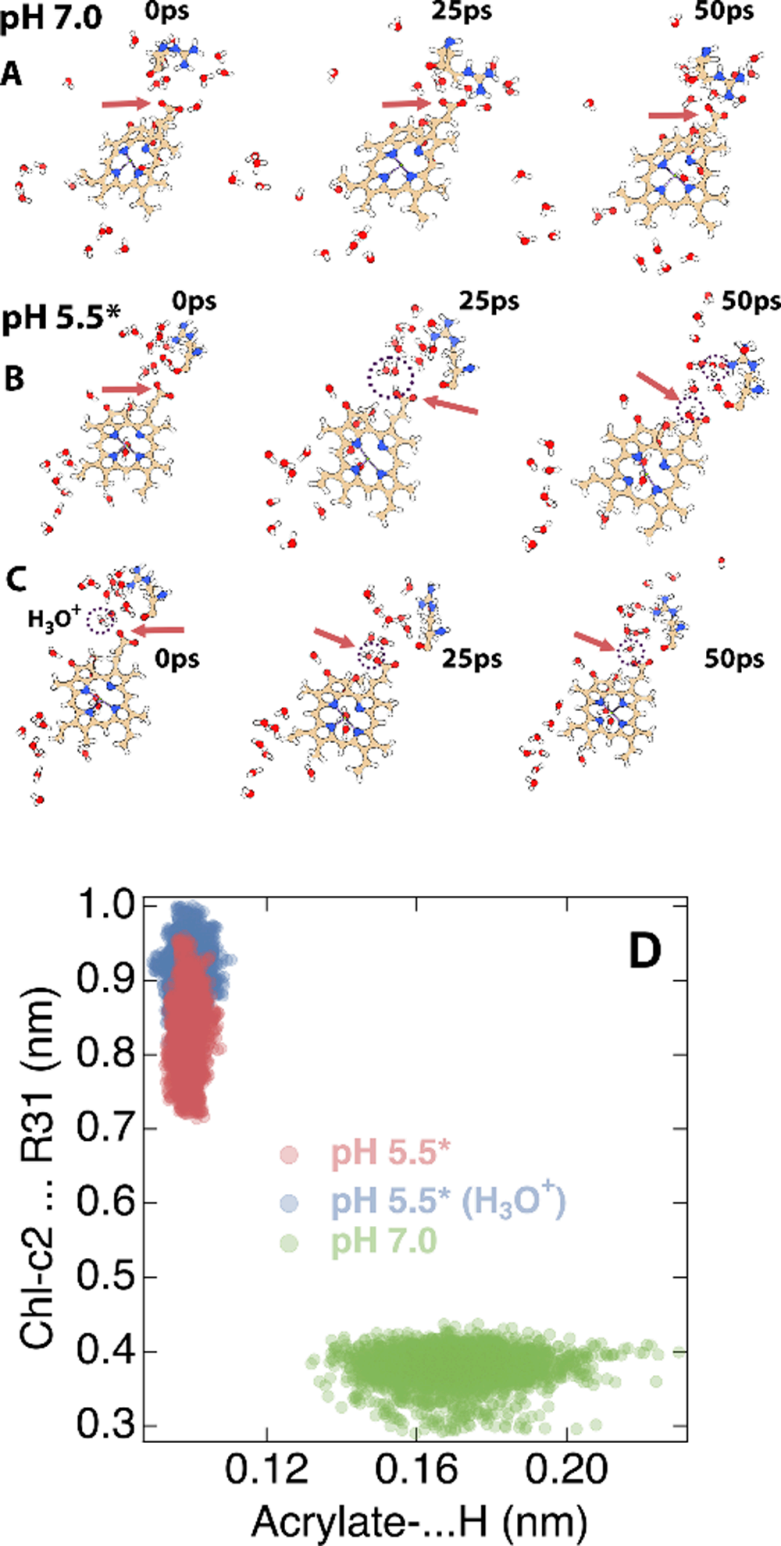
**|** Positions of protons (H+) along with Arg31 (R31) and Chl-c2 observed at different pH values, i.e., at (A) pH 7.0, (B) pH 5.5* in the absence of H_3_O^+^, and (C) in the presence of H_3_O^+^ in the QM region and along the QM/MM MD trajectory at three time points. The protein environment and the lipids have been omitted for clarity. Only Chl-c2, Arg31, and water molecules in a radius of 0.8 nm around the acrylate of Chl-c2 and Arg31 are shown. (De)protonation events are highlighted by circles. Solid red arrows indicate the site of the Chl-c2 acrylate. (D) Distribution of Chl-c2 (acrylate)–proton and Chl-c2 (acrylate)–R31 distance for the different cases in (A), (B), and (C) after the 25 ps time frame.

To further probe the proton transfer mechanism to the Chl-c2 acrylate, we performed density-functional tight-binding (DFTB)-based QM/MM MD simulations of 50 ps each (Figure 8). These simulations have been initiated from the FCP monomer at pH 5.5* and pH 7.0 (three different starting structures, including the median of the most populated cluster of structures in each respective equilibrium trajectory), and the QM region consisted of the deprotonated Chl-c2, Arg31, and all water molecules in a 0.8 nm radius around the acrylate and Arg31. Protonation and deprotonation events are marked by dotted circles in Figure 8A–C for the different protonation states explored. Our findings revealed that the protonation of the acrylate occurs very rapidly with a proton moving along a chain of water molecules in the presence of a nearby H_3_O^+^. Under low lumenal pH conditions, i.e., pH 5.5*, the salt bridge between the acrylate group and the protonated Arg31 weakens, as discussed above (Figure 3A).

A proton transfer to the acrylate takes place even in the absence of H_3_O^+^ through water splitting (−OH, H^+^) within the QM region. Consequently, the OH– group rapidly approaches Arg31. However, under the neutral pH conditions of pH 7.0, the salt bridge between the acrylate and Arg31 is significantly stronger, which discourages proton hopping from water molecules to the acrylate within the time scale probed in the present QM/MM MD simulations. This indicates that at pH 7.0, the possibility for the acrylate to get protonated is lower. This correlation between the acrylate protonation events (acrylate–proton distances) and the acrylate–R31 distance is shown in Figure 8D, for times after 25 ps when proton transfers (if any) have been completed. The QM/MM MD trajectories provide an additional strong hint that protonations at the lumenal side of FCP weaken the acrylate–Arg31 salt bridge and that subsequently the acrylate can get protonated. These simulations strongly suggest that the identified water channel allows the acrylate group of Chl-c2 to get protonated and deprotonated based on the availability or deficiency of excess protons in the water on the lumenal side of the membrane.

### Impact of the pH-Dependent Water Channel and the Switch between Light Harvesting and Photoprotection

**Figure 9:**
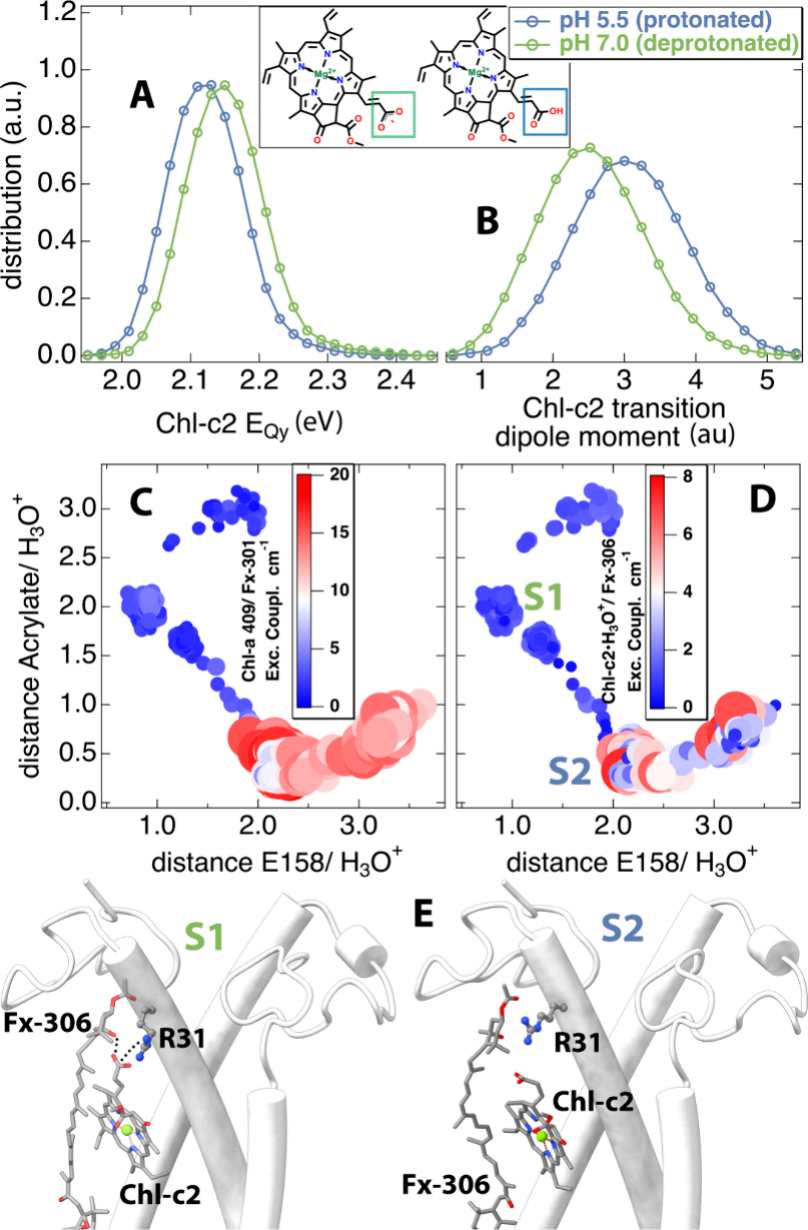
**|** Quenching mechanism. The distributions of Qy excitation energies (A) and transition dipole moments (B) for the protonated (pH 5.5) and deprotonated (pH 7.0) Chl-c2 within the FCP complex along the 1 ns QM(DFTB)/MM MD trajectory. Protonated and deprotonated structures of Chl-c2 are also shown for reference in the inset of (A, B). The excitonic couplings for the Chl-a 409/Fx-301 pigment pair (C) and the Chl-c2/Fx-306 pigment pair (D) along the steered MD trajectory as projected onto two coordinates: the distance of the H_3_O^+^ (oxygen) to (i) the Chl-c2 acrylate (carboxyl group) and (ii) to the Glu-158 (E158) side chain carboxyl group. The sizes of the circles indicate the magnitude of the coupling values, as also indicated by the color code. (E) Representative interactions between the Chl-c2 acrylate, Arg31 (R31), and the ring of Fx-306 for two different states (S1, S2). For reference, the locations of these states are shown in (D) as well.

The pH-dependent water channel is responsible for the protonation of the Chl-c2 acrylate, a finding also supported by the QM/MM MD simulations presented in Figure 8. A H_3_O^+^ can reversibly protonate the acrylate group of Chl-c2 (Figure 9A,B inset). Therefore, it is necessary to characterize both Chl-c2 forms (protonated, deprotonated) at the quantum level upon excitation in energy transfer events. The ground-state geometry of Chl-c2 in the gas phase was optimized at the B3LYP/Def2-TZVP level of the theory. Subsequently, TD-DFT excited-state calculations were performed at the CAM-B3LYP/Def2-TZVP level for the protonated and deprotonated forms. Details of the calculations are provided in Experimental Methods section. The Qy excitation energies and the corresponding transition dipole moments of both Chl-c2 forms are shown in Table 1. Furthermore, we have repeated the same kind of calculations, with the ground state optimized at the DFTB level (Table 1). The optimization of the ground state at this level was performed using the DFTB+ package^80^ on the level of the third-order DFTB theory together with the 3OB-f parameter set.^81,82^ As shown in Table 1, a significant red shift can be observed in the excitation energy of the deprotonated Chl-c2 compared to its protonated counterpart, in both gas phase-optimized structures at the B3LYP and DFTB levels. Moreover, the protonated state exhibits a higher transition dipole moment and oscillatory strength compared to the deprotonated state in both structures. However, the variations in excitation energies and transition dipole moments for structures optimized at the B3LYP level are more noticeable than those optimized at the DFTB level.

**Table 1:** Excitation Energies (Qy in eV) and the Associated Transition Dipole Moments (μ in Atomic Units), and Oscillatory Strength (*f*) of the Chl-c2 Molecule in Both Its Protonated and Deprotonated States at the TD-DFT Level of Theory, for Structures Optimized in the Gas Phase and as Averages for the 1 ns-Long QM(DFTB)/MM MD Trajectory

	**Chl-c2 (protonated)**			**Chl-c2 (deprotonated)**		
	Qy	μ	*f*	Qy	μ	*f*
**B3LYP (gas phase)**	2.262	0.757	0.032	1.649	0.171	0.001
**DFTB (gas phase)**	2.199	0.779	0.033	1.414	0.255	0.002
**QM (DFTB)/MM MD**	2.114	2.944	0.073	2.143	2.430	0.051

Furthermore, we have performed a 1 ns trajectory at the QM/MM MD level in which the QM region is described by the DFTB level of theory and electrostatically linked to the classical AMBER force field. The protonated and deprotonated states of the Chl-c2 molecule are considered as QM regions within a monomeric FCP complex, which is modeled at pH 5.5 and 7.0, as previously described. The resulting distributions of the Qy excitation energies and the transition dipole moments are depicted in Figure 9A,B, respectively. The average excitation energy and transition dipole moment for both protonated and deprotonated Chl-c2 molecules within the FCP complex at pH 5.5 and 7.0 are provided in Table 1. A surprising blue shift of the excitation energy distribution is revealed for the deprotonated Chl-c2 molecule, in contrast to the gas-phase calculations. This finding correlates with a recent QM/MM study reporting a red shift in the protonated Chl-c2 compared to its deprotonated counterpart based on a single QM/MM-optimized structure.^20^ Furthermore, a recent work of our groups emphasized the significance of this shift, indicating that the protonated Chl-c2 molecule exhibits the most pronounced redshift within the pigment network of the FCP complex, attributed to the electric field caused by the protein scaffold.^83^ Moreover, the transition dipole moment of the protonated Chl-c2 has been determined to be larger along the trajectory compared to the gas phase results. This change is, however, not as pronounced as for the gas phase-optimized structure due to the semiempirical DFTB method utilized for ground-state dynamics to account for the electric field effect on Chl-c. These findings are particularly intriguing given that at pH 5.5, the system is expected to be in a quenched state and a higher dipole moment increases the excitonic coupling with adjacent carotenoid molecules.

A pH-dependent presence of water molecules around Chl-c2 or (de)protonation events in its acrylate group can be associated with switching between light harvesting and photoprotection. The Chl-a 409/Fx-301 pigment pair has been implicated in the robust photoprotective mechanism of diatom FCPs.^18,34^ A strong excitonic coupling of Chl-a 409/Fx-301 has been observed for the FCP monomer only in the interaction with the LHCX1 family of proteins in our previous study.^18^ The expansion of the FCP scaffold that is filled with waters (wider channel, state S2, low lumenal pH) is communicated also to the Chl-a 409/Fx-301 pigment pair, only in the dimeric FCP case, as shown by the increased coupling in Figure S6 for the equilibrium trajectories, pushing Chl-409 toward the Fx-301 quencher, in line with previous structural and experimental spectroscopy.^18,34^ The FCP-FCP dimeric interface matches the FCP-LHCX1 interface.^18^ The FCP-LHCX1 interaction has been proved crucial for the activation of the quenching mechanism in diatoms.^84^ Excitonic couplings within FCP could be further increased upon the proton conductance along the identified water channel in FCP. To prove our case, we have calculated the excitonic coupling based on the transition charge from electrostatic potential (TrESP)^85^ between the Chl-c2 and its nearby carotenoids (Fx-306, 303), taking into account the transition charges for both the protonated and deprotonated states of Chl-c2. The TrESP charges for Chl-c2 were determined at the CAM-B3LYP/Def2-TZVP level, whereas for the Fx carotenoid, we employed the DFT/MRCI method. More details are provided in Experimental Methods section. The same approach was employed also for the Chl-a 409/Fx-301 pigment pair. In fact, our steered MD simulations indicate that the movement of H_3_O^+^ along the FCP water channel is correlated with an increase of the excitonic coupling between Chl-a 409 and Fx-301 upon H_3_O^+^ binding to the Chl-c2 acrylate (Figure 9C). Thus, the water channel and the Chl-c2 protonation can be associated with a more efficient excitation energy transfer from Chl-a 409 to the carotenoid Fx-301, leading to energy dissipation as heat.^34^ Additionally, at the conditions for photoprotection, when the lumenal space is acidified, the protonation of the Chl-c2 acrylate, which is possible through the identified water channel, leads to a red shift as shown in Figure 9A, which in turn may affect the efficiency of energy transfer to a nearby quencher carotenoid (Fx-306) or promote the formation of energy traps under photoprotection.^20^ Moreover, the transition dipole moment of the protonated Chl-c2 is significantly larger as depicted in Figure 9B and Table 1, which directly leads to a stronger excitonic coupling to the nearby carotenoid (Fx-306) compared to the deprotonated Chl-c2.

Carotenoids exert short-lived S1/S* states that dissipate excess energy as heat. Recently, it has been reported that the Chl-c2 excitation energy is very sensitive to the electrostatic field in its vicinity. Concurrently, our steered MD runs also indicate an increase of the excitonic coupling between Chl-c2 and the nearby Fx-306 (Figure 9D), but also Fx-303 (Figure S7) upon the binding of H_3_O^+^ to the Chl-c2 acrylate. This binding is directly associated with an increased transition dipole moment calculated for the protonated Chl-c2 as discussed earlier as well as structural changes in the vicinity of Chl-c2 accompanying this binding, with the acrylate losing both hydrogen bond interactions to Fx-306 and Arg31, along with a rotation in the Fx-306 ring (Figure 9E). Substantial changes in the excitation energy flow in the vicinity of Chl-a 409, and Chl-c2, can be expected also due to the changes in the transition dipole moment of Chl-c2 (Figure 9B). Together, these findings indicate that, when the Chl-c2 molecule undergoes protonation, at least the Chl-c2/Fx-306 and Chl-a 409/Fx-301 pairs serve as switches for the excitation energy flow between light-harvesting and photoprotective modes within the FCP complex. The interplay between the excited-state properties of (de)-protonated Chl-c2 and conformational changes is crucial for the switch of the Chl-c2/Fx-306 pair, whereas for the Chl-a 409/Fx-301 pair, conformational changes induced by the water channel primarily control the switch between light harvesting and photoprotection.

A combination or synergy between different sites within FCP (water channel, Chl-c2 acrylate, Chl-c2 site energy fluctuations, Chl-a 409/Fx-301 pigment pair) seems to contribute to the robust and efficient switch between light harvesting and photoprotection in the diatom *P. tricornutum* diatom.

## Conclusions and Outlook

In this study, we have run extensive molecular simulations for FCP monomers, dimers, and tetramers at varying pH values and the results are supported also by QM/MM-MD simulations. We propose that in the light-harvesting state (neutral lumenal pH, transthylakoid ΔpH = 0), the Chl-c2 acrylate is not accessible from the two aquatic phases and remains deprotonated (state S1, Figures 2A and 4C). A strong interaction between the Chl-c2 acrylate and Arg31 is found, as indicated by short distances in this pair (Figure 3A). When the lumenal space is acidified, selected lumenal residues get protonated. These residues trigger conformational changes in the FCP protein scaffold that result in an expansion of a putative water channel within the FCP protein with access to the lumenal space, which in turn is filled by water molecules (states S2–S4, Figures 3A and 4C). This expansion enables the access of more water molecules mainly to the Chl-c2 acrylate/Arg31 that weaken the associated Chl-c2–Arg31 salt bridge and in turn enable an effective protonation of only the Chl-c2 acrylate via the water channel upon sudden changes in the lumenal pH (acidification under photoprotection). This result correlates with the partial protonation of the Chl-c pigments with a ratio of 3:1 within FCPs.^33^ The identified water channel does not disrupt the buildup of an enhanced proton gradient (ΔpH) across the thylakoid membrane. This is achieved by a mechanism similar to the one found in aquaporins. The direction of the water molecules within the channel is reversed roughly around the position of the Chl-c2 acrylate, along with a desolvation penalty for H_3_O^+^ at the same region. At low lumenal pH, immobile waters are present only at the stomal side of the FCP complex, blocking the neutral aquatic phase of the stroma to reach the Chl-c2 acrylate. We provide a direct connection of (i) the protonation of key residues at the lumenal side of the FCP protein upon photoprotection, (ii) the water flux in the identified putative water channel connecting the lumenal space with the Chl-c acrylate, and (iii) the acrylate protonation to the transition between the light-harvesting and the quenching states of the major FCP complex. The pH-dependent dynamics of the water/proton channel correlate with the excitonic couplings within key pigment pairs in FCP: the Chl-a 409/Fx-301 and the Chl-c2/Fx-306. This finding forms the basis for future research especially concerning the downregulation of photosynthesis in diatoms. We have to note that in the Cryo-EM structure of the PSII-FCP supercomplex of the diatom species *Chaetoceros gracilis*, Chl-cs are also present however exposed to the lumenal side of FCP,^23^ which would enable the pH-dependent (de) protonation of Chl-c2 in this species directly, without the need for a water channel to be formed, as lumenal pH changes upon the transition from light harvesting (neutral pH) to the quenching state (pH 5.5). Future simulations will also address these FCPs from the diatom *C. gracilis* and compare the results to the FCP dynamics in *P. tricornutum* and probe the role of LHCX1 on the formation of the water channel/proton conductance. The diversity of the light-harvesting complexes of diatoms and the identification of an unexpected water channel therein are indicative of the strong abilities of the diatoms to successfully survive under various light and environmental conditions; abilities that make diatoms a dominant and adaptive photosynthetic species in the oceans. The downregulation of photosynthesis is a promising research target for crop improvement and biomass increase.^87−89^ By modifying the number of proteins involved in photoprotection, a quick recovery back to the light-harvesting state can be achieved, which in turn avoids that excitation energy is lost, i.e., is dissipated as heat.^90^ Moreover, the fundamental research on photoprotection can form a potential basis for artificial photosynthesis.^91^ A thorough conformational sampling of the FCP proteins and the water (proton) dynamics along the whole water channel among different diatom species will be at the focus of a future combined quantum mechanics/metadynamics investigation.^92−95^

## Experimental Methods

### Setup of FCP Models

The crystal structure of the fucoxanthin chlorophyll a/c–binding (FCP) protein from the diatom *P. tricornutum* (pdb: 6a2w)^16^ has been used for the initial coordinates to build the FCP monomer model. The dimer was built with the orientation of the monomers as defined in the same crystal structure of Wang et al.^16^ For the tetramer, the FCP monomer in the structure of Wang et al. was superimposed with the tetramer chains AB, CB, ZA, and BB with the best crystallographic resolution (high quality score) in the FCP-PSII supercomplex from the diatom *C. gracilis* (pdb: 6jlu).^21^ Thus, the tetramer in the FCP-PSII supercomplex was used only as a scaffold to transform the Wang et al. structure into a tetramer. Moreover, the Amber ff14sb force field^96^ was employed for the polypeptide chains. In all FCP models, the carotenoids Fx and Ddx were described by Amber compatible parameters.^96−98^ The chlorophyll a/c molecules were based on parameters from refs (99,100). The all-atom models, as defined previously, were embedded in membrane patches of around 350 thylakoid lipids^101,102^ described by the Amber force field.^96^ The content in thylakoid lipids was adopted from the model thylakoid membrane in ref (18); 45% monogalactosyl-diacyl-glycerol (MGDG), 25% digalactosyl-diacyl-glycerol (DGDG), 25% sulfo-quinovosyl-diacyl-glycerol (SQDG), and 5% phosphatidyl-glycerol (PG) in line with an increased content of the negatively charged SQDG-PG lipids (30%) compared to the plant thylakoids (15–20%).^103^ The MGDG-DGDG lipid content is at 70% to simulate high-light adapted diatoms compared to low-light grown diatoms (50%).^103^ Around 25000 TIP3P^104^ water molecules have been used to fully hydrate the system. The six models contained 150 mM KCl, with a surplus of K^+^ ions to neutralize both the protein charges and the negatively charged thylakoid lipids in each system. The equilibrated unit cell dimensions of each model were 10.3 × 10.3 × 11.5 nm^3^ (monomer) as well as 11.2 × 11.2 × 10.4 nm^3^ (dimer, tetramer).

### Classical Molecular Dynamics

Based on published protocols, all models have been relaxed and equilibrated with gradual removale of constraints on the heavy backbone atoms of the protein.^18,102^ In a series of constant volume NVT, and constant pressure NPT ensembles, the temperature was increased from 100 to 310 K prior to the production runs. For the production classical Molecular Dynamics (MD) simulations, Newton’s equations of motion are integrated with a time step of 2.0 fs. The leapfrog integrator in GROMACS 2021^105^ was employed. The production runs have been performed in the constant pressure NPT ensemble with semi-isotropic couplings in the xy membrane plane and in the *z*-direction (compressibility at 4.5 × 10^–5^ bar^–1^). Moreover, the van der Waals interactions were smoothly switched to zero between 1.0 and 1.2 nm with the Verlet cutoff scheme. Electrostatic interactions were truncated at 1.2 nm (short-range) and long-range contributions were computed within the particle mesh Ewald (PME)^106^ approximation. All hydrogen–heavy atom bond lengths were constrained employing the LINCS algorithm.^107^ The v-rescale thermostat^108^ (310 K, temperature coupling constant 0.5) and the Parrinello–Rahman barostat^109^ (1 atm, pressure coupling constant 2.0) have been employed. Independent trajectories (replicas) were initiated from different structures extracted 10 ns apart from the last stage of the equilibration. All additional parameters are identical to those in published work for the FCP monomer.^18^ The total simulation time sums to 24 μs for the FCP models (four independent trajectories of 0.5 μs per tetramer models, four independent trajectories of 0.5 μs per dimer models, eight independent trajectories of 0.5 μs per monomer models × 3 pH-protonation states). The total simulation time is distributed per monomer sampling within the tetramer, dimer, or monomer, which translates to 3 pH states × (4 monomers × 2 μs + 2 monomers × 2 μs + 1 monomer × 4 μs) = 48 μs sampling for the FCP monomer in the different multimeric states. The first 100 ns from each trajectory was considered as further equilibration, and the analysis was only performed for the final 400 ns of each independent trajectory. Structures were collected every 1.0 ns for all the trajectories. Shorter trajectories were also run with a sapling every 10 ps for the nonequilibrium dynamics, with otherwise identical parameters as the longer ones.

### Steered MD Runs

The steered MD run was performed in the constant pressure NPT ensemble with semi-isotropic pressure coupling (298 K, 1 atm). The same parameters as in the classical MD runs were used; however, a time step of 1 fs was set to integrate the Newton’s equations of motion, because of the flexible H_3_O^+^ model employed. H_3_O^+^ was steered across the FCP scaffold by biasing its distance to Glu-158 (lumen), Chl-c2 acrylate, and the stromal space (center of mass of a triad of acidic residues Asp-129, Glu-130, and Glu-131).

### Markov State Model

The FCP protein scaffold was extracted, without protons, water, and ions from the independent production trajectories beyond 100 ns. We additionally dismantled the dimers–tetramers into monomer trajectories. In total, we end up with 112 independent trajectories (48 μs) that describe the dynamics of the FCP monomer protein scaffold. Trajectory frames were taken every 1 ns. The frames in all trajectories were structurally aligned on the same reference initial structure, by fitting on the Cα atoms with PyMOL 2.5 (Schrödinger, L., & DeLano, W.) to ensure consistency in the analysis. Furthermore, the PyEMMA package in Jupyter notebooks was employed.^110^ Only the torsional angles of the FCP residues 25–56 (helix a1), 75–83 (helix a1′), 84–103 (helix a2), and 129–161 (helix a3) were selected as initial input features for model construction based on the residue numbering in the FCP structure by Wang et al.^16^ The MSM uncertainty bounds were computed using a Bayesian scheme. We found that the slowest implied time scales converge quickly and are constant within a 95% confidence interval for lag times above 50 ns. The validation procedure is a standard approach in the MSM field.^36−38^ A lag time of 85 ns was selected for Bayesian model construction, and the resulting models were validated by the Chapman–Kolmogorov (CK) test. Both the convergence of the implied time scales and the CK test confirm the validity and convergence of the MSM. A lag time of 85 ns and three tICA eigenvectors (dimensions) were thus chosen based on the VAMP2 scores,^111−113^ to identify a set of the slowest modes among all the initial input features,^114^ and for dimensionality reduction. The modes constitute a linearly optimal combination of input features, which maximizes their kinetic variance. The conformations of the FCPs were projected on these slowest modes as defined by the tICA method, and then the trajectory frames were clustered into 100 cluster centers (macrostates) by *k*-means clustering, as implemented in PyEMMA. The resulting macrostates (*k*-means) were further coarse grained into a smaller number of four metastable states or macrostates using PCCA++ as implemented in PyEMMA. The optimum number of macrostates (four) was proposed based on the VAMP2 score.

### QM/MM Calculations

50 ps-long QM/MM MD simulations have been performed using the DFTB+/GROMCAS interface.^115^ The QM region comprised the deprotonated Chl-c2, Arg31, and the water molecules within an 0.8 nm radius around Chl-c2 and Arg31. For the MM region, we employed the Amber FF^96^ as in the classical MD to describe the remainder of the system. Notably, to separate Arg31 from the surrounding protein environment, we introduced link atoms at the C–O bonds. Three sets of simulations were conducted: two under acidic conditions at pH 5.5* and one under neutral pH conditions, all with acrylate initially in its deprotonated form. For the simulations at pH 5.5*, we tested two scenarios: one with an H_3_O^+^ ion in the quantum mechanics (QM) region and another without. In case of the simulations at neutral pH, no H_3_O^+^ ions were present. The QM/MM simulations were initiated at the median structures of the most populous cluster of structures for each pH case along the respective equilibrium trajectories.

#### Excited-State Calculations and Excitonic Coupling

The optimization of the ground state for Chl-c2 in the gas phase was performed at the DFT level (B3LYP/Def2-TZVP) followed by TD-DFT calculations (CAM-B3LYP/Def2-TZVP) for the excited states. Moreover, 1 ns QM/MM MD trajectories were initiated at both pH 5.5 and 7.0, at the same TD-DFT level (CAM-B3LYP/Def2-TZVP). The ORCA quantum chemistry package^116,117^ was utilized to perform these computations. Additionally, we have utilized the Tamm–Dancoff approximation (TDA) and the resolution of identity (RI) approximation to accelerate the TD-DFT calculations, employing the RIJCOSX method for Coulomb integrals and Hartree–Fock exchange, alongside def2/J as an auxiliary basis set, all within the ORCA program. The RI approximation was also utilized for the B3LYP/Def2-TZVP level of theory to optimize the Chl-c2 molecule in the gas phase. The trajectory frames were stored at a time step of 1 ps, resulting in a total of 1000 frames. Subsequently, TD-DFT calculations were performed on these conformations in a QM/MM scheme at the CAM-B3LYP/Def2-TZVP level of theory for the excited-state calculations.

The excitonic coupling between Chl-2/Fx-306 and Chl-a 409/Fx-301 were carried out based on the TrESP method, as reported in detail elsewhere.^85^ The transition charges for (de-)protonated Chl-c2 were extracted at the TD-DFT level employing the Multiwfn program,^118^ whereas for the Fx carotenoids, the charges were taken from our previous study at the DFT/MRCI level.^18^ Moreover, we have considered the absolute magnitude of the coupling since the rate of exciton transfer (*k*) ∼ exciton coupling (|*V*|^2^). The transition charges for the deprotonated Chl-c2 were employed for the calculation of exciton couplings up to the point where H_3_O+ is within a distance less than 0.5 nm from the Chl-c2 acrylate at the steered MD trajectory, and then we switched to the transition charges for the protonated Chl-c2 for the calculation of the excitonic couplings between Chl-c2 and Fx-306.

## Supporting Information Available

Free energy surface by Markov state modeling; Chl-c1 to Lys-31 distance distributions; radial distribution functions of waters around Chl-c1; radial distribution functions of oxygen and nitrogen atoms of water, protein, and lipids around H_3_O^+^; H_3_O coordination network across the protein scaffold; pH-dependent excitonic couplings for the Chl-a 409/Fx-301 pigment pair; and excitonic coupling for the Chl-c2/Fx-303 pigment pair (PDF)
