# Supplementary material for: An Unexpected Water Channel in the Light-Harvesting Complex of a Diatom: Implications for the Switch between Light Harvesting and Photoprotection
Source: ACS Phys Chem Au. 2024 Aug 21;5(1):47–61. doi: 10.1021/acsphyschemau.4c00069 (PMC11758497; doi:10.1021/acsphyschemau.4c00069)
Supplement: Supplementary file 1 — pg4c00069_si_001.pdf [file pg4c00069_si_001.pdf]

Supporting Information for

An unexpected Water Channel in the Light-Harvesting Complex of a Diatom: Implications for the switch between light harvesting and photoprotection

Vangelis Daskalakis,*[1§] Sayan Maity,[2§] and Ulrich Kleinekathöfer[2]

[1] Department of Chemical Engineering, University of Patras, Caratheodory 1, University Campus, GR 265 04 Patras, Greece.

[2] School of Science, Constructor University, Campus Ring 1, 28759 Bremen, Germany

* Corresponding author, e-mail: vdaskalakis@upatras.gr, Tel (work): +30 2610997820, ORCID: 0000-0001-8870-0850

[§]Authors VD and SM have equally contributed.

Contents

Figures S1 – Free Energy Surface by Markov State Modeling

Figure S2 – the Chl-c1 to Lys-31 distance distributions

Figure S3 – Radial Distribution Functions of waters around Chl-c1

Figure S4 – Radial Distribution Functions of oxygen and nitrogen atoms of water, protein, and lipids around H_3O+

Figure S5 – the H3O coordination network across the protein scaffold

Figure S6 – the pH dependent excitonic couplings for the Chl-a 409/ Fx-301 pigment pair

Figure S7 – the excitonic coupling for the Chl-c2/ Fx-303 pigment pair

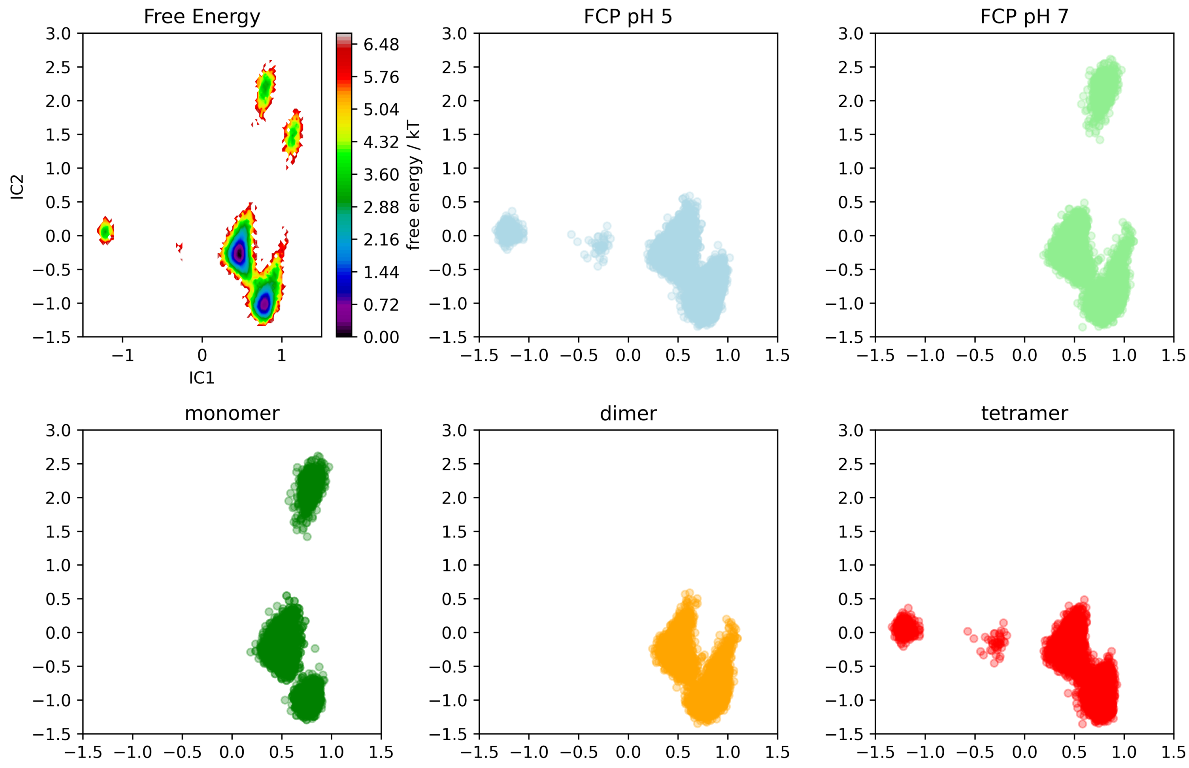

Figure S1 | Weighted free energy surfaces of the FCP protein along with the positions of the sampled points in the different FCP models on the same free energy space spanned by the tICA coordinates IC1 and IC2). The energy values are given in k_BT, with k_B being the Boltzmann constant and T being the temperature.

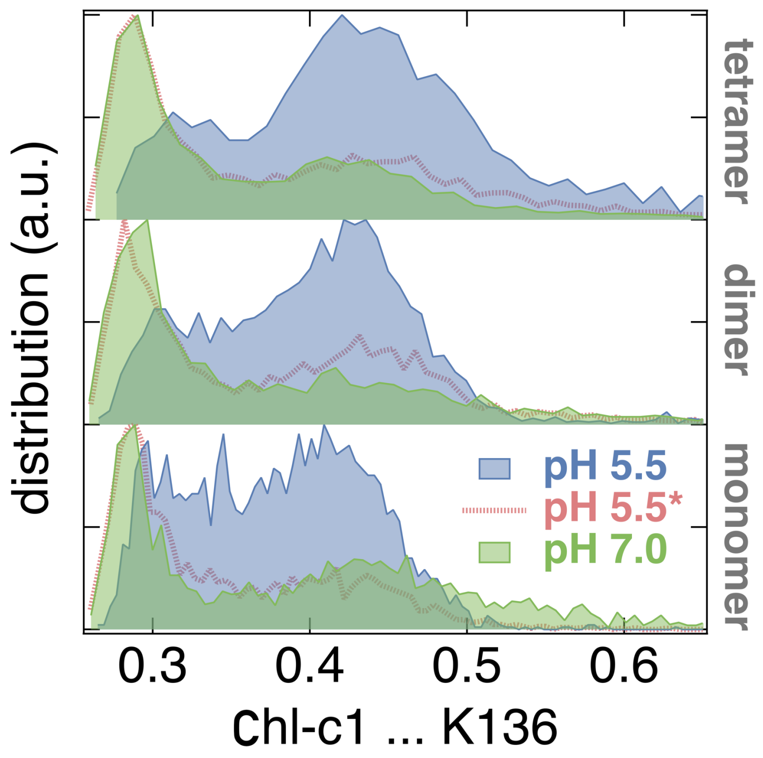

Figure S2 | Distributions of the distances between the chl-c1 acrylate and Lys-136 (L136) for the different FCP states.

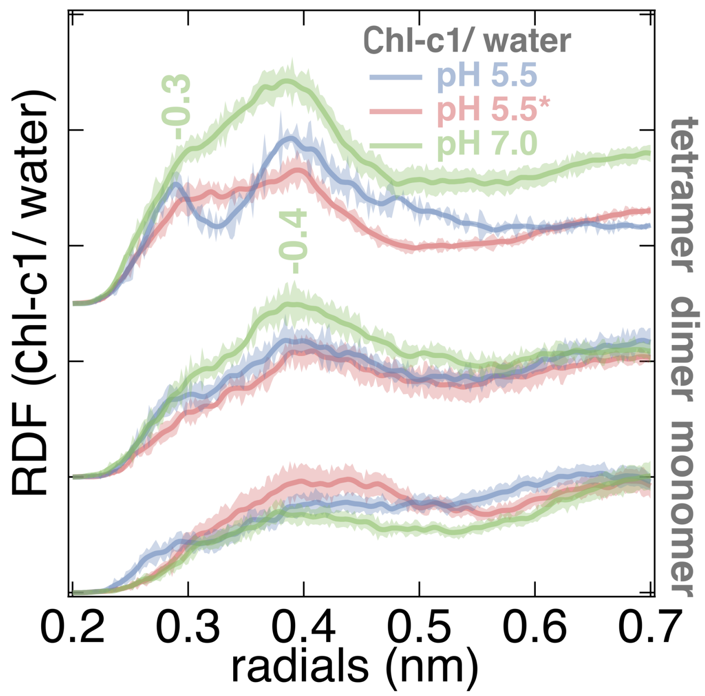

Figure S3 | The radial distribution functions (RDFs) of the water molecules around chl-c1 acrylate, averaged over 500ns windows and the monomers in each dimer, tetramer trajectory. The colored shaded areas represent the standard deviations out of the averaging.

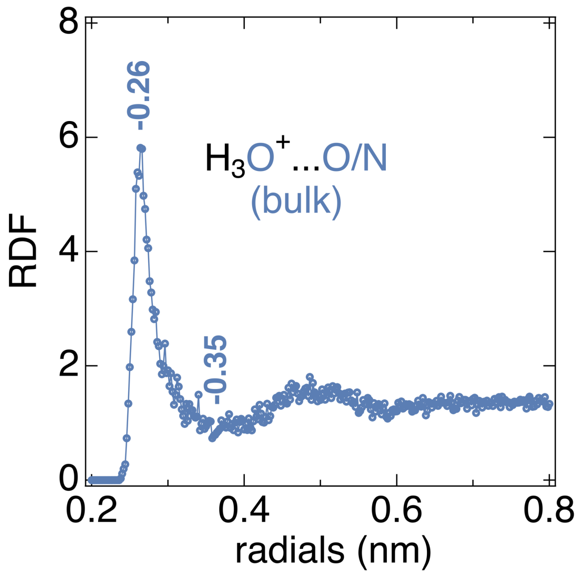

Figure S4 | The Radial Distribution Function (RDF) of oxygen and nitrogen atoms around the H_3O^+ oxygen is shown. This has been calculated for the H_3O^+ at equilibrium runs (100ns), where the hydronium ion is moving between the protein-lipid-water interfaces and the bulk water, never entering the channel.

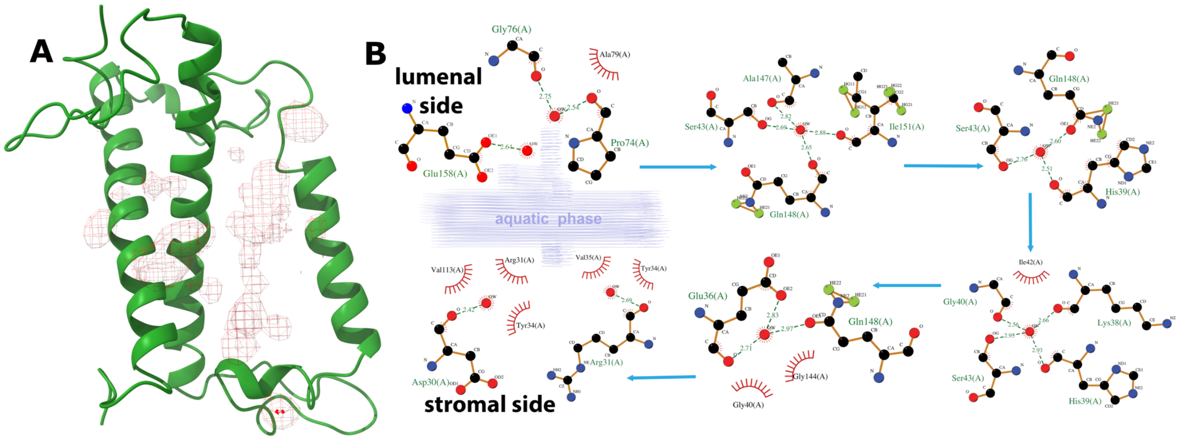

Figure S5 | A. Density of the H_3O^+ sampled positions in the RAMD trajectories shown as red wireframes. The density was calculated by the VolMap Tool in VMD, averaged over all RAMD frames and visualized by Chimera 1.8 at the 0.001 level. **B.** The hydrogen bonding network for the H_3O^+ across the FCP protein scaffold; from the lumen to the stroma. The H_3O^+ is shown as red rayed spheres and labeled "OW". The aquatic phase of the lumenal and stromal sides are sketched for reference. The hydrogen bonding network has been calculated by LigPlot+ 2.2.8. Blue arrows indicate the different H_3O^+ coordination states in the direction from lumen to stroma. Eyelash-like sketches indicate unfavorable interactions.

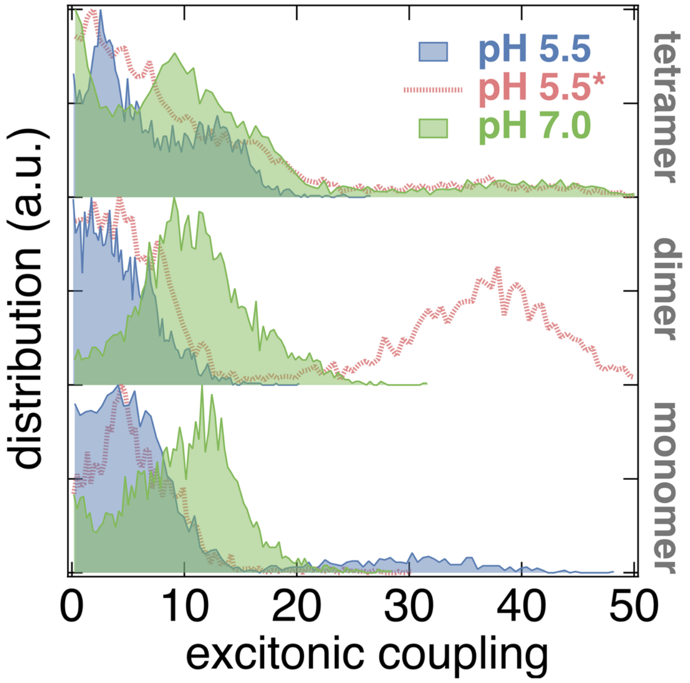

Figure S6 | Quenching mechanism. The distribution of excitonic couplings for the Chl-a 409 / Fx-301 pigment pair along the equilibrium MD trajectories at different pH values and oligomerization states of FCP.

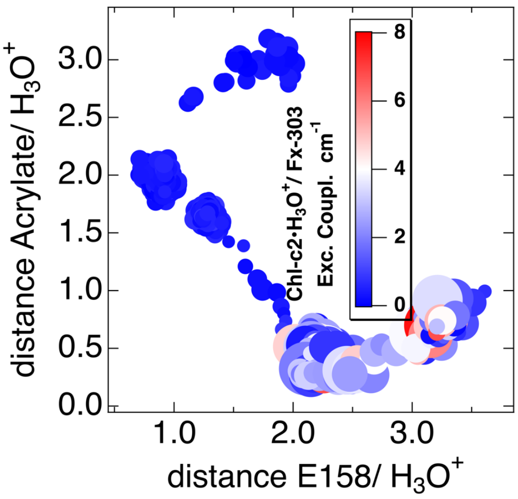

Figure S7 | Quenching mechanism. The excitonic couplings for the Chl-c2/ Fx-303 pigment pair along the steered MD trajectory as projected onto two coordinates: the distance of the H_3O^+ (oxygen) to (i) the Chl-c2 acrylate (carboxyl group) and (ii) to the Glu-158 (E158) side chain carboxyl group. The sizes of the circles indicate the magnitude of the coupling values, as also indicated by the color code.
